# New design of operational MEMS bridges for measurements of properties of FEBID-based nanostructures

**DOI:** 10.3762/bjnano.15.103

**Published:** 2024-10-23

**Authors:** Bartosz Pruchnik, Krzysztof Kwoka, Ewelina Gacka, Dominik Badura, Piotr Kunicki, Andrzej Sierakowski, Paweł Janus, Tomasz Piasecki, Teodor Gotszalk

**Affiliations:** 1 Department of Nanometrology, Wrocław University of Science and Technology, Janiszewskiego 11/17, 50-370, Wrocław, Poland; 2 Institute of Microelectronics and Photonics, Łukasiewicz Research Network, Lotników 32/46, 02-668, Warsaw, Poland

**Keywords:** FEBID, MEMS, MEMS bridge, nanowires, opMEMS

## Abstract

Focused electron beam-induced deposition (FEBID) is a novel technique for the development of multimaterial nanostructures. More importantly, it is applicable to the fabrication of free-standing nanostructures. Experimenting at the nanoscale requires instruments with sufficient resolution and sensitivity to measure various properties of nanostructures. Such measurements (regardless of the nature of the quantities being measured) are particularly problematic in the case of free-standing nanostructures, whose properties must be separated from the measurement system to avoid possible interference. In this paper, we propose novel devices, namely operational micro-electromechanical system (opMEMS) bridges. These are 3D substrates with nanometer-scale actuation capability and equipped with electrical contacts characterised by leakage resistances above 100 GΩ, which provide a platform for comprehensive measurements of properties (i.e., resistance) of free-standing FEBID structures. We also present a use case scenario in which an opMEMS bridge is used to measure the resistance of a free-standing FEBID nanostructure.

## Introduction

Nanoelectronics is the fastest developing branch of modern electronic technology. Reduced dimensions allow for lower power consumption of the circuit and higher operating speeds [1–2]. Even more advantages (e.g., developed surface or reduced capacitance) are brought by volumetric, self-standing electronic nanostructures, which provide an experimental basis for their own properties and can serve as building blocks for nanoscale devices, in which phenomena such as giant piezoresistivity, single-electron tunnelling, or field emission occur [3–5].

There are only a few processes with resolution and repeatability suitable for creating nanostructures, and even fewer are available for self-standing nanostructures. That includes epitaxial techniques as well as focused electron beam-induced deposition (FEBID) [6–7]. Integration of the nanostructures into larger devices remains one of the major challenges, especially when non-silicon materials (such as carbon nanotubes or multimaterial nanowires) are used [8–10]. Among the fabrication methods, particle beam-based methods offer the greatest versatility for rapid prototyping and experimentation, such as electron and ion beams, and in particular FEBID [11–13].

FEBID has a resolution of a few nanometres [14] and can be automated [15] or combined with other technologies [16–17]. It can be used to deposit conductive or insulating layers, as well as layers of special materials [18–20]. The limitation of FEBID technology is proximity deposition, also known as the halo effect, where secondary electrons cause unwanted deposition in an area (the halo) significantly larger than the assumed beam spot [21]. If the deposited material is electrically conductive, it can cause leakage, that is, parasitic current flow through the insulating layer.

**Figure 1:**
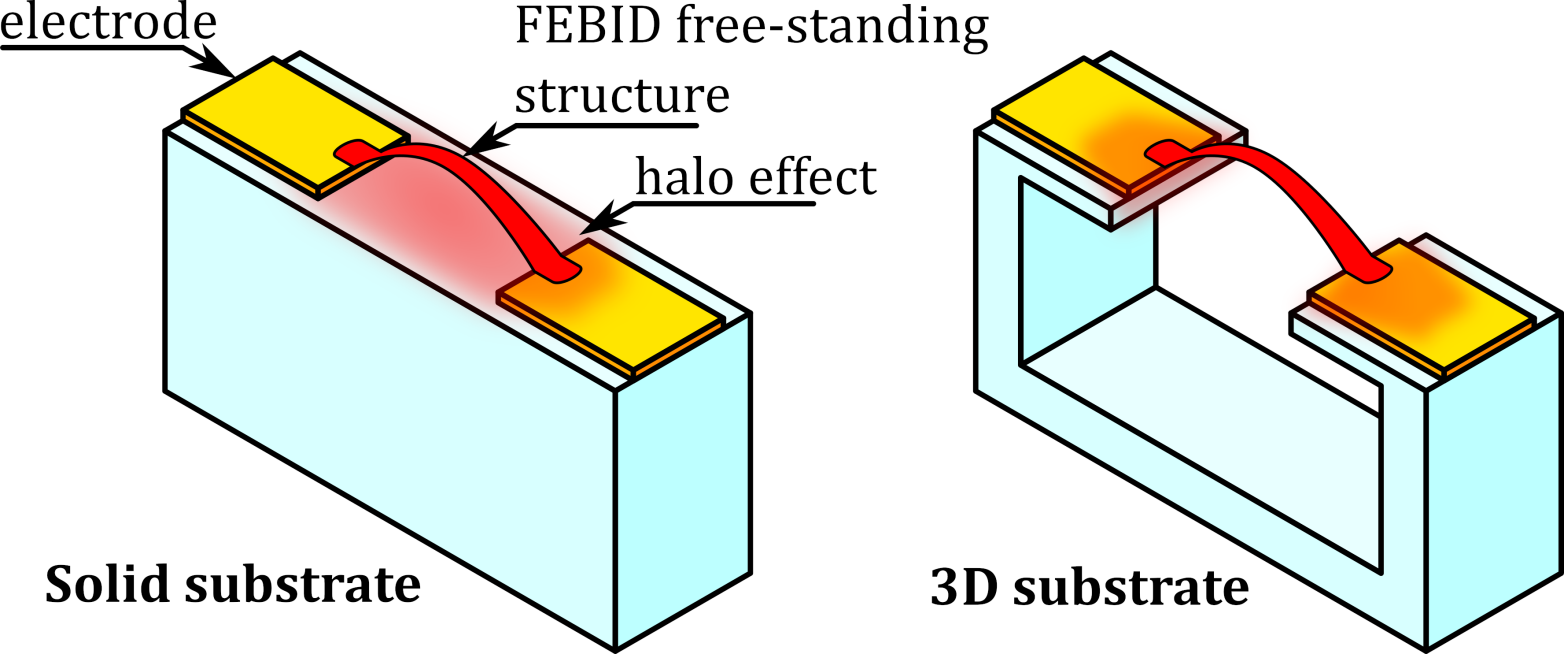
Visualisation of the halo effect under a freestanding nanostructure on a solid and 3D substrate.

Eliminating the halo effect is virtually impossible; however, the size of the halo can be measured, and its negative effects can be eliminated by proper spacing between conducting FEBID deposits [22]. Another approach is to fabricate 3D substrates (Figure 1) with gaps that interrupt the halo deposition [23], as we have done in our previous work [24]. There are ongoing investigations related to the fabrication, characterization, and application of flat nanostructures [25], but in the flat architecture, there are less possibilities to determine the leakage currents. Moreover, existing methods are detrimental to the proper operation of the device [26–27].

Such 3D substrates can be realised as micro-electromechanical system (MEMS) measurement platforms, which have the advantage of achieving controlled displacement through an actuation mechanism. This opens the possibility to perform both electrical and mechanical measurements of nanostructures, in particular, free-standing nanostructures, which are the most interesting for their mechanical and electrical properties combined in nanoscale phenomena [28–30].

The requirements for the desired MEMS measurement platforms are as follows: They should be dimensionally compatible with the nanostructure to be tested and allow for actuation with an embedded actuation mechanism. To operate, MEMS platforms require auxiliary equipment such as a scanning electron microscope (SEM) or electrical measurement equipment. The experiments performed in this way belong to the so-called lab-in-SEM (LiS) technologies.

The technology for MEMS fabrication comes from microelectronic surface and bulk micromachining methods [31]. MEMS dimensions are typically defined by photolithography and e-beam routines combined with dimensionally limited wet and dry etching processes.

**Figure 2:**
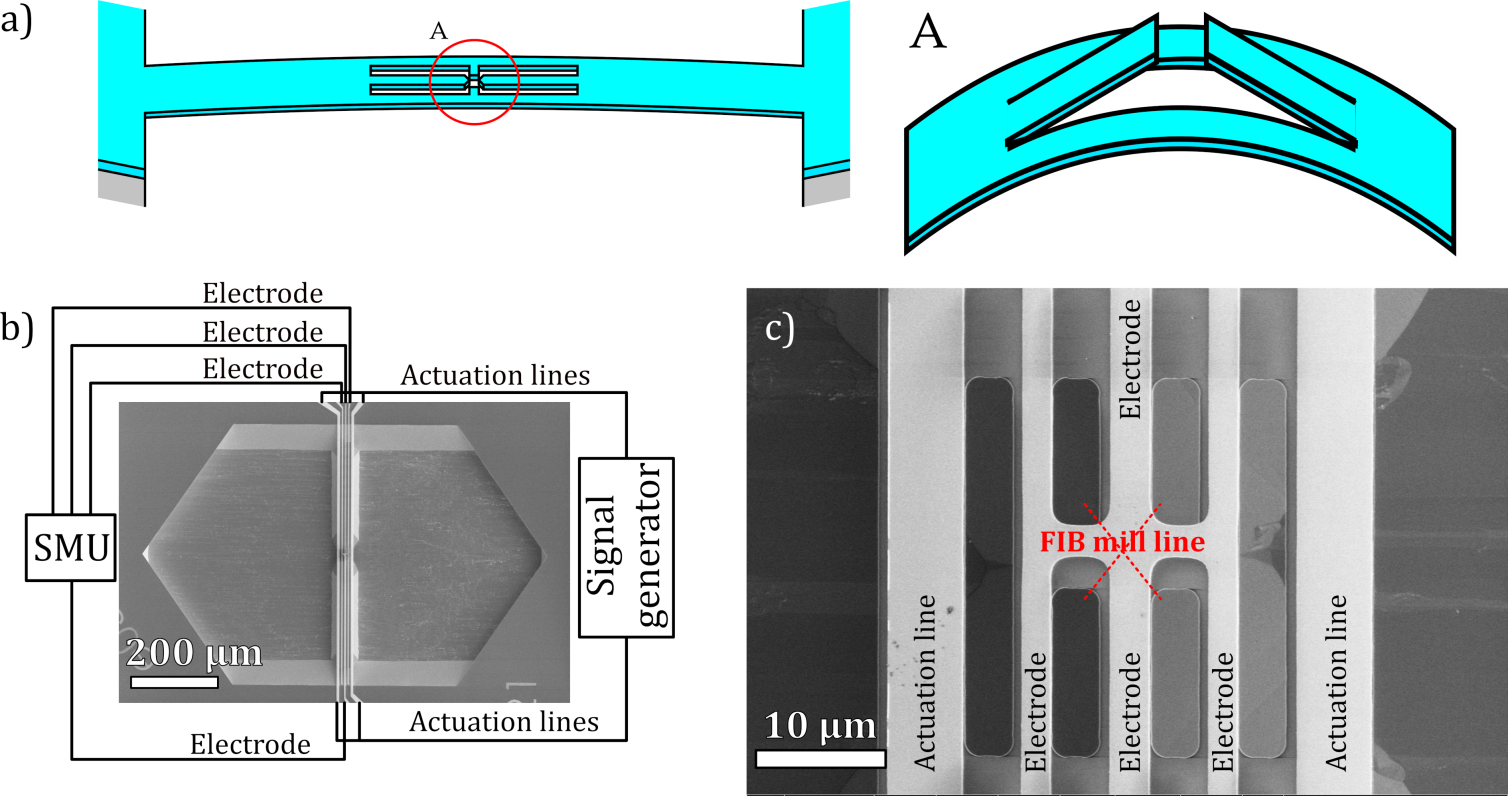
MEMS bridge shown (a) schematically with RoI formed (left) and RoI distance geometry (right), (b) on a SEM image as a whole with schematic electrical connections, and (c) on a SEM image focused on the RoI, with path descriptions and ion beam milling lines marked.

The most advanced MEMS allow for movement in all six degrees of freedom (DOFs), while the simplest are defined in only one DOF [32]. The latter include so-called MEMS bridges formed as double-clamped beams [33], which have been shown to work as traceable displacement generators [34]. When a MEMS bridge is cut in the middle to form two separate cantilevers (Figure 2a), the two coincident edges create a region of interest (RoI) where the distance between the cantilevers is a function of the structure deflection (Figure 2a). However, parasitic strain can cause unforeseen static changes in the geometry of the RoI. This problem is addressed by focused ion beam (FIB) technology, which can be used as a strain engineering tool.

We will refer to MEMS with a formed RoI as operational MEMS, or opMEMS for short. In contrast to previous solutions involving the embedding of nanostructures in MEMS [35], opMEMS combine the ability to induce motion in the RoI with the possibility of connecting electrical signal paths for direct electrical measurements.

In this article, we present the design and operation of opMEMS for measurements of FEBID nanostructures. We describe the basic parameters of the opMEMS bridge as a mechanical resonator. We show how the actuation can be used to control displacements with picometre resolution. We present how the intrinsic strain can be described by appropriate modelling and controlled through nanomodification. Finally, we show results of resistance measurements of a free-standing FEBID nanostructure deposited across the RoI of a LiS-embedded opMEMS bridge.

## Materials and Methods

### Design of opMEMS bridges

The opMEMS were fabricated on an undoped ⟨110⟩ silicon substrate on which a 40 nm silicon nitride (Si_3_N_4_) layer was deposited via CVD. The 40 nm thick platinum paths were then patterned by lift-off photolithography. The opMEMS bridge body was defined photolithographically with a feature size of 2 µm, etched by dry oxygen plasma etching (DRIE) and then released by KOH anisotropic wet silicon etching. The silicon edges under the opMEMS bridge bases were defined to be vertical and precisely located, thanks to the lattice orientation. The opMEMS were 600 μm long, 100 μm wide, and 80 nm thick, with 40 nm of silicon nitride body and 40 nm of platinum paths on top. To reduce the stiffness of the opMEMS bridge and facilitate wet etching underneath, openings were made in the silicon nitride layer near the bases.

**Figure 3:**
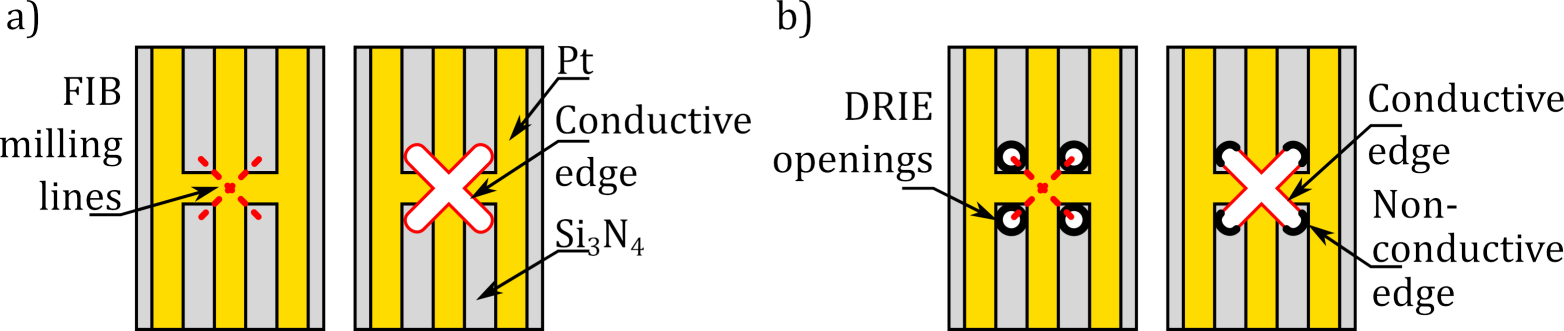
Influence of DRIE-milled openings on current leakage across the slit edge. (a) Conducting paths are electrically shorted by leakage along the edge of the FIB-milled slit. (b) The edge of the FIB-milled slit is not continuous; therefore, paths are not electrically shorted.

The designed bridge contained three conductive measurement lines and two actuation lines (Figure 2c), with the measurement paths joined at the centre of the bridge. The RoI slit was formed using the gallium FIB to mill a 200 nm wide slit (Figure 2c). As a side-effect, gallium was implanted along the edge of the RoI slit, so to prevent the leakage current; additional openings were formed using the DRIE process to create a non-conductive edge of the slit (Figure 3b).

### Mechanical characterization of opMEMS bridges

As mentioned in [36], MEMS are well suited for electrothermal actuation. The actuation process is driven by the Joule heat from a current flowing through the actuation lines of an opMEMS bridge, which causes a change in temperature and, in turn, a displacement resulting from a mismatch in the thermal expansion coefficients of the opMEMS materials (Pt: 8.9 µm/mK, Si_3_N_4_: 3.3 μm/mK) [37]. Joule heat is dissipated in the actuation lines (Figure 2b,c), which then causes transverse bending of the whole opMEMS bridge.

The opMEMS bridges are also susceptible to electromagnetic actuation [38], but this technique is difficult to apply in SEM chambers (the ultimate working environment for opMEMS) because of the difficulty of obtaining a controlled magnetic field inside.

In the experiments, the resonance of the opMEMS was measured outside the vacuum chamber using a SIOS nano vibration analyser to assess the properties of the active device. The measured level of vibration of the MEMS bridge at a known temperature allows its stiffness to be determined with an accuracy of 5% [38].

First, the thermomechanical noise of the structure vibration was measured, then the displacement of the electrothermally actuated opMEMS bridge. A sinusoidal actuation signal was generated by a Tektronix AFG3021B signal generator and conditioned by a self-designed current source. The current amplitudes did not exceed 3 mA.

### Finite element method modelling

The finite element method (FEM) was used to investigate the structural effects present in the opMEMS bridges. Of particular importance was the presence of eigenstrains [39], which would affect the shape of the RoI after FIB milling. Eigenstrains can occur due to mismatches between the crystalline lattices of the deposited materials or due to interactions between materials, but they can also be thermally induced. Only the latter can be simulated with the chosen FEM engine, but such models have already been developed [40–41] and should also be considered in the advanced numerical model.

The analysis of internal strain in opMEMS starts with the assumption of a residual strain field induced by processing steps. The concept of eigenstrain is flexible enough to include all residual stresses in solids [42]. This is done using pseudo-thermal strain, which induces an arbitrary eigenstrain field, which is then compared with experimental data [43]. The aim of the simulation was to recreate the strain state in an attempt to fully understand it and to find countermeasures for the resulting spacing in the RoI.

The opMEMS were designed and modelled using the FEM software Comsol Multiphysics 6.1 (licence no. 17078442). In the simulation, opMEMS bridges were modelled using the material parameters provided by the “MEMS” library. The geometry of the model was defined by the technological design. Microfabrication artefacts were not taken into account. The model was analysed in the steady-state study. The solid mechanics and heat transfer modules were used together with the thermal expansion multiphysics module. The following constraints were imposed: no displacement and no heat sink on the substrate surfaces. Heat sources were defined on the actuation paths as temperature sources of alternating temperatures. The heat source temperature was 293 K (given by the experimental conditions). The initial and heat sink (ambient) temperatures were varied from ambient to 200 K above ambient (493 K) to simulate manufacturing conditions. Gravity was defined proportional to the local mass. No additional forces or initial displacements were defined.

### LiS experiments

The combination of ion and electron columns forms a dual-beam system, which is an efficient tool for nanoprototyping [44]; hence, LiS experiments were performed using a Helios NanoLab 600i dual-beam system from FEI (Hillsboro, Oregon).

SEM offers a theoretical imaging resolution better than 2 nm. Accordingly, SEM allows not only for the visual assessment of the shape and dimensions of a structure, but also for the observation of movement and deflection of an opMEMS. At the same time, the ion beam allows for local doping of the substrate and anisotropic milling. The NanoLab 600i also provides three gas injection systems (GIS) for the supply of precursors required in the FEBID processes. In the experiments carried out, trimethyl(methylcyclopentadienyl)platinum(IV) (MeCpPtMe_3_) was used to deposit the Pt(C) material. Microfabrication of nanostructures and modification of opMEMS, both mechanically and electrically, is thus possible with a resolution of tens of nanometres.

FIB processing has an influence on the intrinsic strains of the structures; therefore, it can be used as a strain engineering tool [45]. When a suitable substrate material is used, a localised strain concentration can influence the shape of the device, as has been shown in the case of FIB origami [46]. More recently, Masteghin et al. have shown how strain engineering and eigenstrain modification can influence single-material MEMS devices [47]. The exact mechanism depends on the ion and substrate materials, but surface amorphisation or material implantation can occur [45,48], leading to the development of a strained layer. All in all, FIB straining becomes an indispensable tool in the case of opMEMS.

The Helios NanoLab 600i was supplemented with additional measuring equipment. A sample connector was placed inside the vacuum chamber. Coaxially protected signals were fed through feedthroughs to the Keithley SMU 2634B and function generators. The instrumentation was controlled by proprietary software developed in the Department of Nanometrology, Wrocław University of Science and Technology.

## Results

**Figure 4:**
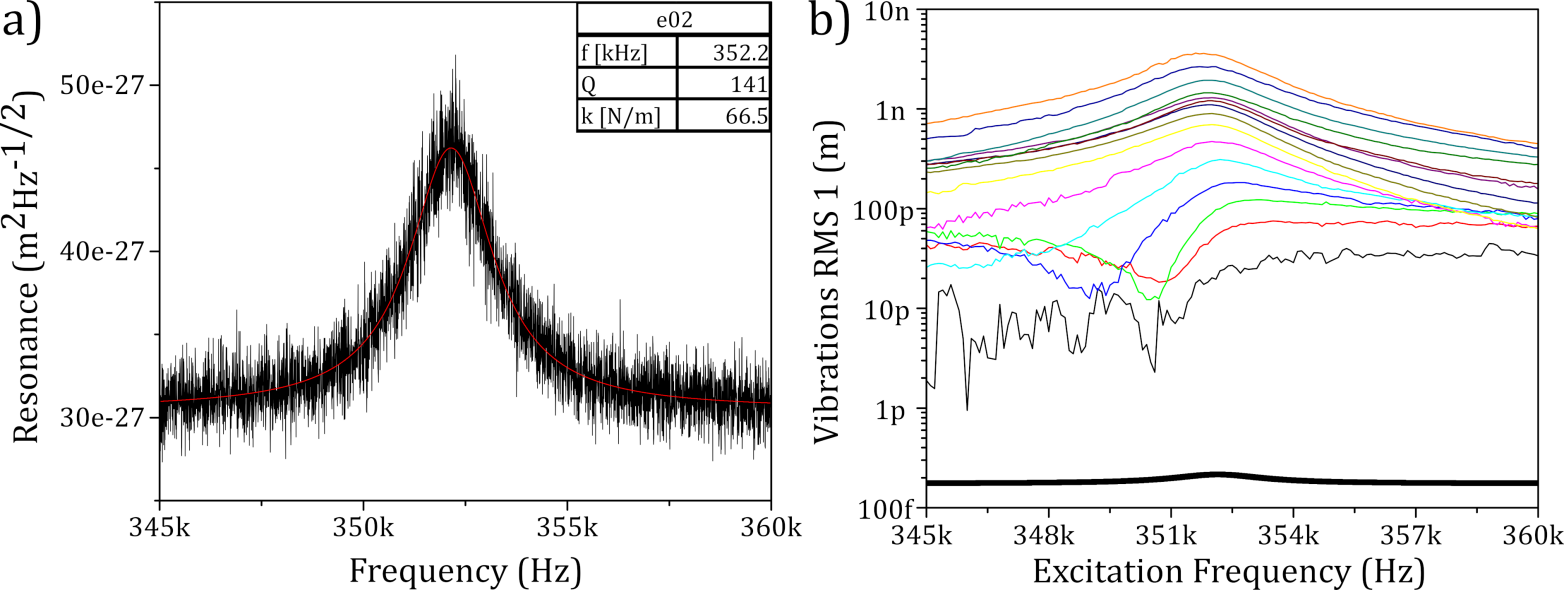
Oscillation periodograms of opMEMS with the peak related to the resonant frequency at 352 kHz recorded for (a) thermomechanical self-induced oscillations and (b) electrothermally forced oscillations for currents between 200 μA and 3 mA with 200 μA steps. The measurable resonant deflection is below 30 pm, limited by the current noise. Thermomechanical noise has been included for reference (black line).

First, opMEMS bridges were characterized using spectral analysis of vibrations [49]. The thermomechanical noise analysis approximates the bridge as a simple harmonic oscillator with one DOF, as has been used for determining the spring constant of AFM cantilevers [50]. This approach provides an approximation for the stiffness *k* in terms of the measured thermally induced displacement *z* and temperature *T*,


[1] $$k=\frac{k_{\text{B}}T}{\langle z^{2}\rangle },$$

where *k*_B_ is the Boltzmann constant. Spectral analysis also provides the other basic parameters such as resonant frequency *f* and Q-factor (Figure 4).

The displacement of the electrothermally actuated opMEMS bridge was recorded for currents with amplitudes ranging from 200 μA (lower limit of the current source used in the experiment) to 3 mA (the assumed limit). It has been shown that achievable actuation deflections are as low as 28 pm. However, the deflection is limited by the noise of the actuation signal, which can be observed as a shift in the noise floor for the smallest deflections. In addition to the above parameters, the resistances of the actuation and measurement lines were measured to be 340 and 450 Ω, respectively.

**Figure 5:**
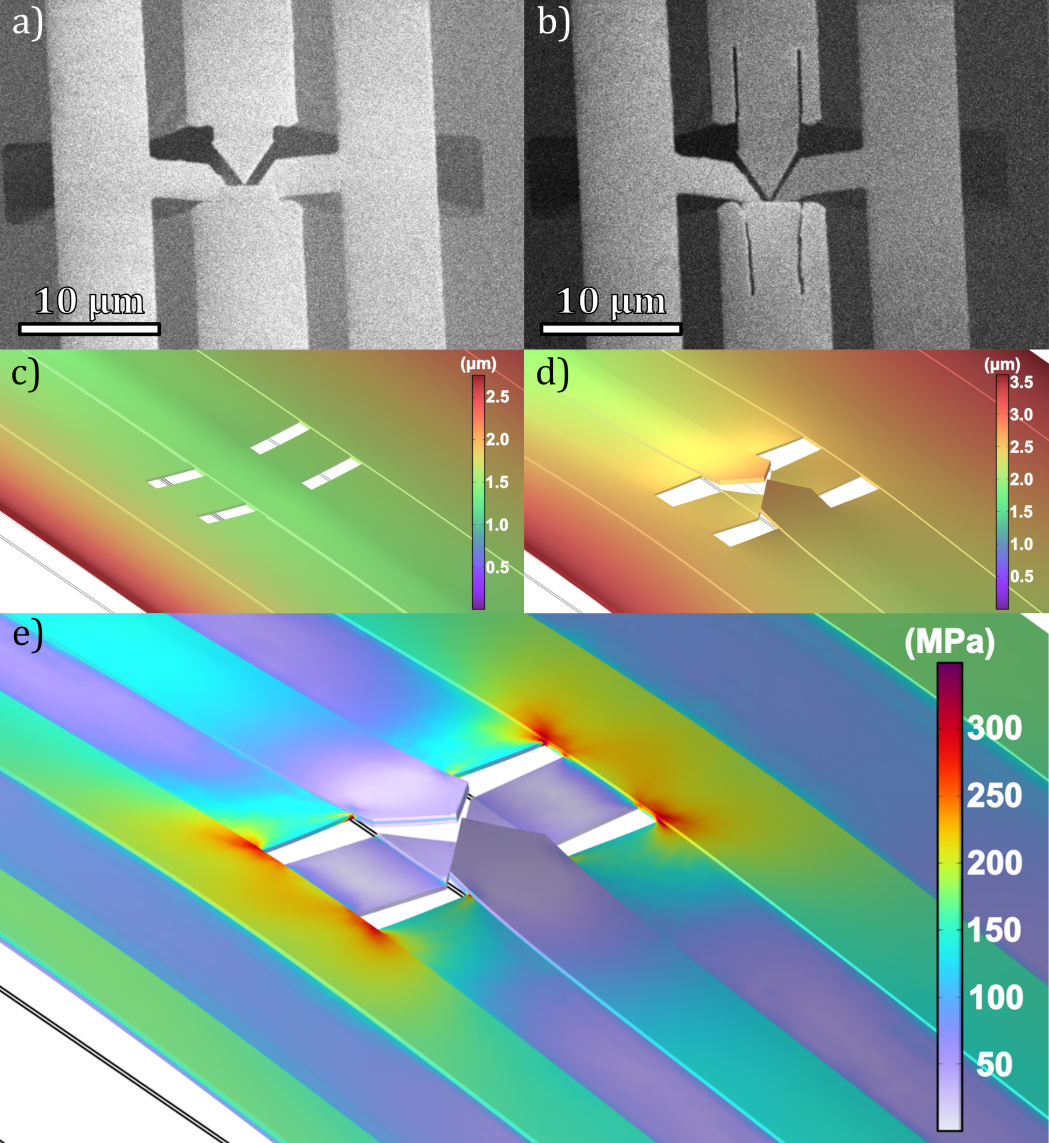
RoI of an opMEMS (a) before and (b) after FIB milling, imaged with SEM at 52° angle of incidence, with a rough value of the distance in the RoI indicated. Cantilevers in the RoI have their visible edges surrounded by a solid line and their invisible edges surrounded by a dashed line. Panels (c) and (d) show the magnitude of displacement (in μm) from FEM simulations for cases before and after FIB milling at 393 K (100 K above ambient), respectively. (e) Von Mises strain (in MPa) around the formed RoI, showing concentration of strain around edges and near the cantilevers, indicating areas suitable for strain reduction.

The opMEMS bridge was then milled to obtain four coincident electrodes in the RoI (Figure 5) with the following FIB parameters: energy 30 keV, current 99 pA, 1 μs dwell time, 10.5 nm pitch, 7500 passes, and beam orthogonal to the surface. Due to the effect of residual strain, the RoI was formed with large (over 1.5 μm) internal spacing (Figure 5). The strain model developed with FEM showed that the strain was the result of tension along the cantilevers in the RoI. The pseudo-thermal strain corresponding to the experimentally obtained strain was the result of the sink temperature of 393 K.

In the experiment, we observed that gallium FIB with 30 keV energy directed at the RoI bends the cantilevers downwards by changing the strain in the top layer of the structure [47]. In the FEM simulation, the presence of the gallium irradiated layer was accounted for by replacing the top 15 nm of the platinum layer with a material of lower thermal expansion coefficient. In addition, the simulation showed that introducing slits along the cantilevers in the direction of the bridge axis (i.e., elongating the cantilevers) reduces the residual strain and, consequently, the distance in the RoI.

**Figure 6:**
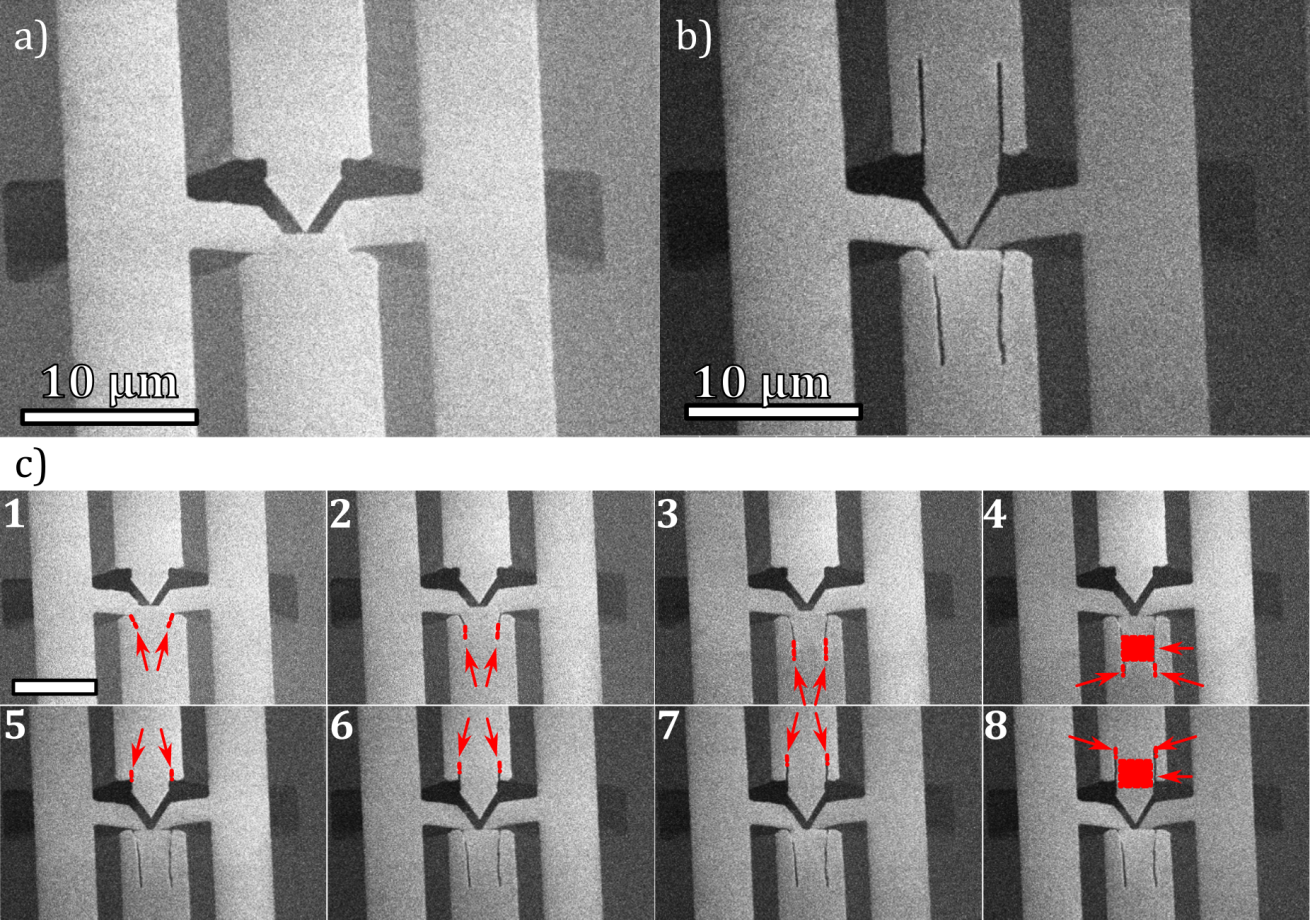
RoI of an opMEMS (a) before and (b) after FIB alignment. The series in panel (c) shows consecutive steps of the alignment process, where each step is a new milling (red lines) and irradiation process (red field shows irradiated area) with a total dose of 500 nC/nm^2^. The scale bar in (c) is 10 μm long.

The alignment of the cantilevers in the RoI was therefore a combination of slit milling and FIB straining of the cantilevers with a total dose of 500 nC/nm^2^ (Figure 6). These two steps were repeated alternately until the spacing in the RoI was reduced to less than 100 nm, as confirmed by SEM imaging (Figure 6). Understanding FIB-induced strain as a physical phenomenon would require a repeatable experimental setup to collect data on the influence of different FIB parameters. An example of such a setup would be the cantilever described in [43].

**Figure 7:**
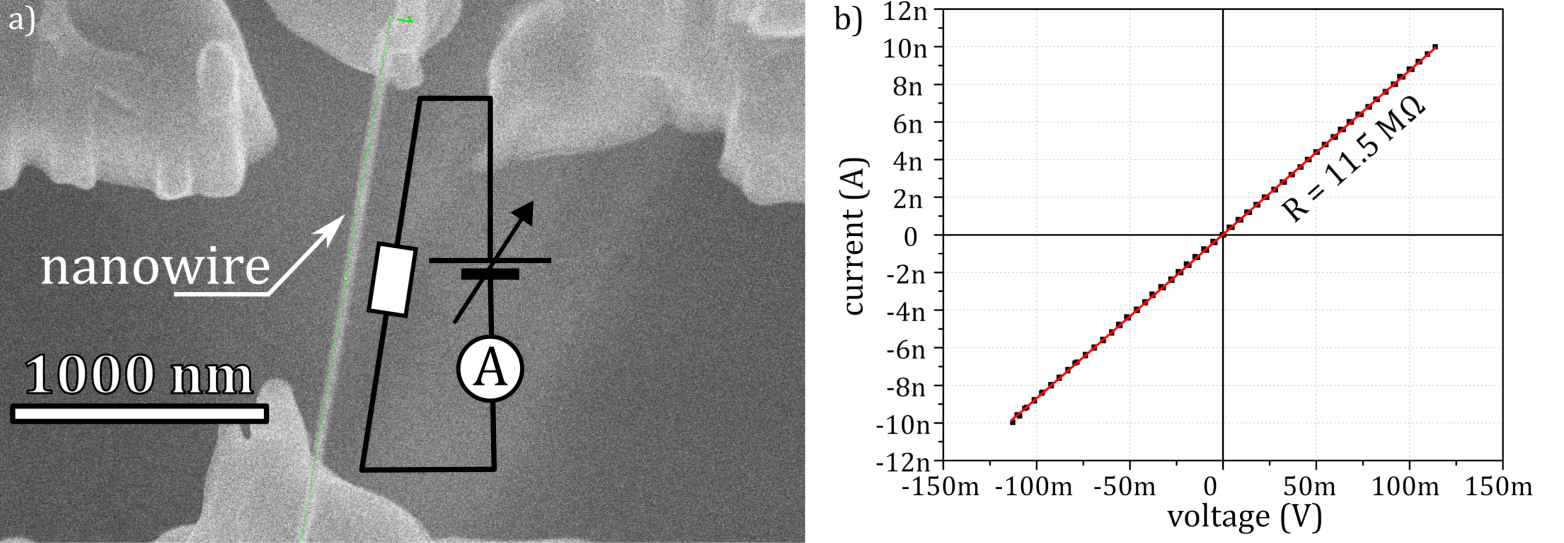
Resistance measurements of a free-standing nanowire deposited in the RoI of an opMEMS bridge. (a) Nanowire deposited in the RoI of the opMEMS with electrical measurement setup depicted and (b) *I–V* curve of the resistance measurement of the nanowire with currents varying between −10 and 10 nA.

**Figure 8:**
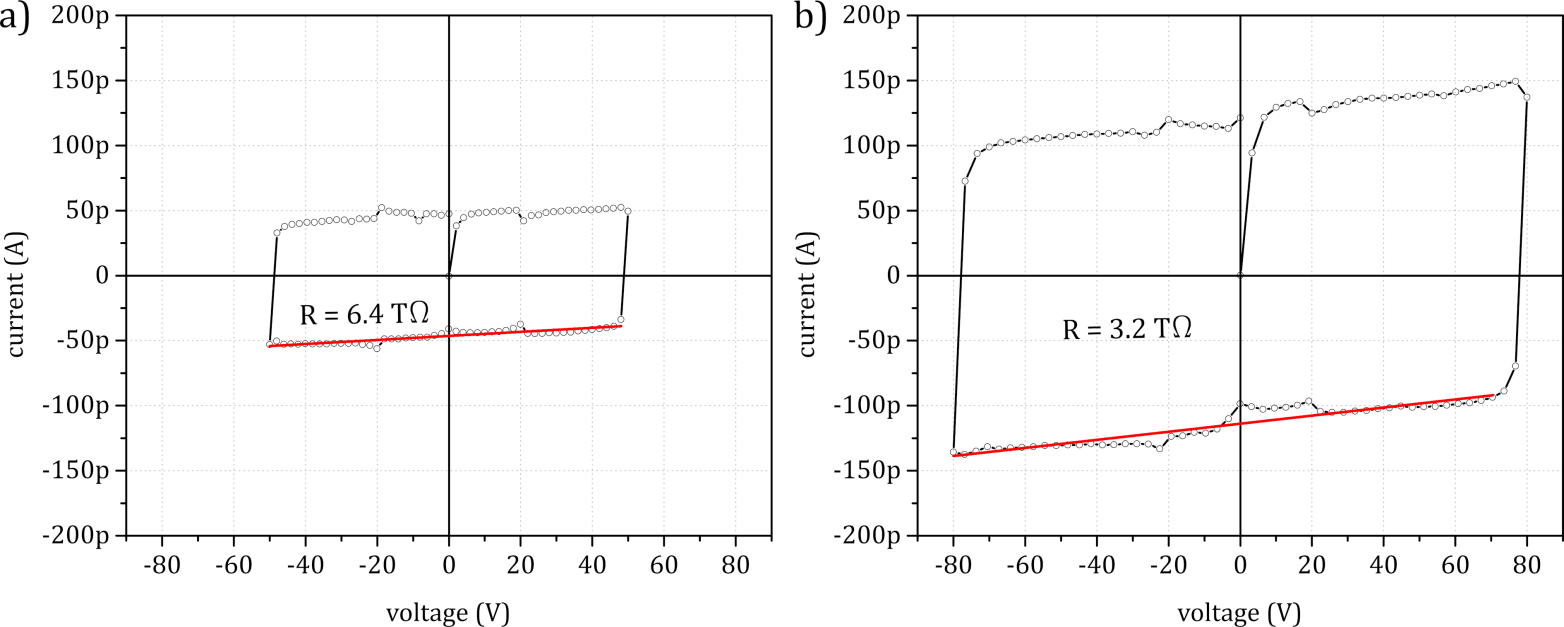
*I*–*V* curve of an opMEMS bridge (a) after gallium FIB milling of the slit in the RoI and (b) after removal of the measured nanowire. In both cases the resistance is above 100 GΩ, and the *I*–*V* curves represent the capacitance charging current.

On the base of the aligned opMEMS, a FEBID nanowire was deposited (Figure 7a) in a single-step process, where point-by-point deposition was performed along the pattern line using the following parameters: 5 keV, 17 pA, 13 ms dwell time, a single line pattern in a single pass with 4 nm pitch, and MeCpPtMe_3_ precursor at 321 K with a flow rate of 8.5 × 10^−4^ μm^3^/s. The pattern line was set to begin on one electrode and end on the other (Figure 7). In between, the nanowire growth proceeded horizontally with an apex by choosing appropriate dwell time and pitch for the deposition. The dimensions of the nanowire were 60 nm in diameter and 1.6 μm in length. The junction was tested by resistance measurements (Figure 8b). The measured resistance was 12 MΩ, giving a resistivity of approximately 2.1 Ω·cm, which is consistent with the reported resistivity of the FEBID material [24].

The resistivity was also measured across the milled structure before nanowire deposition (Figure 8a) and after FIB removal (Figure 8b). The results obtained show that in both cases the measured current was mostly related to the charging and discharging of parasitic capacitance. Voltages higher than 80 V were not used in order not to exceed the device breakdown voltage.

## Discussion

In the measurements described, opMEMS proved to be a suitable platform for FEBID nanostructure metrology. It has been shown that it is possible to deposit a free-standing nanostructure in the form of a single FEBID nanowire, to make electrical contacts and to perform electrical measurements of this nanostructure.

It is worth noting that 3D substrates in the form of MEMS bridges allow sub-nanometric actuation with resolution down to tens of picometres (the resolution of actuation is limited by the background noise of 200 fm/Hz^0.5^), which is a promising feature for future applications of opMEMS as an experimental platform, for example, for studying quantum phenomena such as electron tunnelling or field emission, which are exponentially dependent on the distance in the RoI [51–52]. Precise distance control is also a promising mechanism for gate control in single-electron devices [53].

The RoI distance resulted as an effect of eigenstrain relaxation in the newly formed structure, similar to the eigenstrain evaluation technique described in [54]. At this point, the precision of distance control is limited by the opMEMS eigenstrain, which can be effectively mitigated by stress engineering and FIB modifications. These methods are efficient for experimental work, but there is also an opportunity to improve opMEMS by modifying the geometry that leads to stress relief. For the more complex opMEMS, FEM simulation should be used to solve the design problems. The experiment we conducted proves that it is possible to eliminate structurally induced eigenstrains in the RoI. Successful modification was achieved using an iterative approach.

Only a single ion dose implantation value was used, as the aim of the modification was not the exact determination of the FIB influence on the surface strain. However, with exact knowledge of this influence, the modification would become a predictable, even automatable process. The complex structure of the opMEMS bridge is not well suited for quantitative analysis of the FIB strain process. Instead, for a comprehensive analysis of FIB-induced strain, the analysis should be performed on a model MEMS device, similar to our previous work [17] or the method developed by the authors of [55], who analysed residual stresses in thin films by deflecting a cantilever of defined size from a uniform membrane. We see a need for such experiments for future improvement of our proposed RoI spacing tuning method.

The proposed approach allowed us to evaluate the leakage currents separately from the nanodevice properties. It was shown that the leakage was not affected by gallium FIB milling or conductive FEBID deposition in the vicinity of the RoI. This is particularly important in the field of high-resistivity materials (e.g., FEBID) where the elimination of parasitic interactions is critical to the measurement.

More importantly, our opMEMS already integrates four measurement lines, with the ability to expand to six or eight separate electrodes in the RoI. This encourages us to think of opMEMS as an experimental platform for more complex measurements than a simple two-line connection case. In the future, we will pursue multipoint measurements of nanomaterials in search of their electrical or mechanical properties.

## Data Availability

All data that supports the findings of this study is available in the published article and/or the supporting information to this article.
